# Synthesis of Alkyl Aryl Sulfones via Reaction of *N*-Arylsulfonyl Hydroxyamines with Electron-Deficient Alkenes

**DOI:** 10.3390/molecules22010039

**Published:** 2016-12-28

**Authors:** Yunhui Bin, Ruimao Hua

**Affiliations:** Department of Chemistry, Tsinghua University, Beijing 100084, China; yunhuibin@gmail.com

**Keywords:** alkyl aryl sulfones, electron-deficient alkenes, *N*-arylsulfonyl hydroxylamines

## Abstract

Alkyl aryl sulfones were prepared in high yields via the reaction of *N*-arylsulfonyl hydroxylamines with electron-deficient alkenes. These reactions have the advantages of simplicity, easily available starting materials and mild reaction conditions.

## 1. Introduction

**Figure 1:**
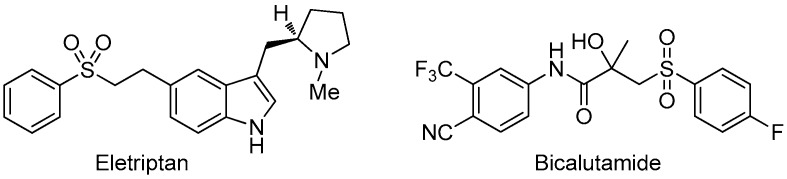
Examples of pharmaceutical molecules with structural unit of alkyl aryl sulfone.

**Scheme 1:**
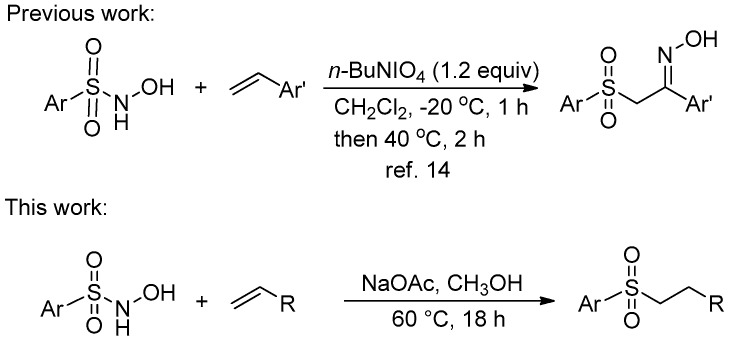
Synthesis of alkyl aryl sulfones from *N*-arylsulfonyl hydroxylamines with alkenes.

Sulfones are important starting compounds in the synthesis of different sulfur-containing compounds and natural products [1]. Sulfones also exist in a wide range of biologically active molecules, such as oxycarboxin (a highly efficient agricultural fungicide) [2], fipronil (a pesticide) [3], and benzobicyclon (a herbicide) [4]. In addition, the structures of alkyl aryl sulfones are well known as the core structural unit in pharmaceutical molecules. For instance, eletriptan is an efficient drug for early migraine [5], and bicalutamide is used for the treatment of advanced prostate cancer [6] (Figure 1). Therefore, a variety of synthetic approaches have been developed for the synthesis of alkyl aryl sulfones, including the Zn/CuI-mediated coupling reaction of alkyl halides with vinyl arylsulfones [7], FeCl_3_/TMSCl-catalyzed β-sulfonation of α,β-unsaturated carbonyl compounds with *p*-toluenesulfinates [8], the aerobic oxysulfonylation of alkenes with various sulfinic acids [9], Cu(OAc)_2_-catalyzed direct oxysulfonylation of alkenes with dioxygen and sulfonylhydrazides [10], the reaction of alkenes with sodium arylsulfinates catalyzed by molecular iodine under aerobic oxidative conditions [11], sulfonylation of activated alkenes with sulfonylhydrazides [12], the direct sulfonylation of 2-methylquinolines with sodium aryl sulfinates mediated by KI in the presence of oxidant [13], nickel-catalyzed hydroxysulfonylation of alkenes using sodium sulfinates [14], and oxygen-mediated reaction of diethyl 1-arylvinyl phosphates with arylsulfinic acid [15]. Recently, He and co-workers reported the preparation of α-sulfonylethanone oximes from the reaction of styrenes with substituted *N*-arylsulfonyl hydroxylamines in the presence of tetrabutylammonium periodate as oxidant (*n*-Bu_4_NIO_4_) (Scheme 1) [16]. In this paper, we report the reaction of *N*-arylsulfonyl hydroxylamines with electron-deficient alkenes to provide an alternative procedure for the synthesis of alkyl aryl sulfones under mild conditions (Scheme 1).

## 2. Results and Discussion

We initiated our study on the reaction of *N*-phenylsulfonyl hydroxylamine (**1a**) with methyl acrylate (**2a**) to optimize the reaction conditions. As summarized in Table 1, when a mixture of **1a** and **2a** (2.0 equiv.) in CH_3_CN was heated with stirring at 60 °C for 18 h, not any product was formed by the analyses of the reaction mixture by GC-MS, and both **1a** and **2a** were recovered (entry 1). However, in the presence of NaOAc (1.0 equiv.), repeating the same reaction gave the desired compounds **3aa** in 39% GC yield (entry 2) [17], indicating that the presence of base is crucial for the formation of **3aa**. In addition, when CH_3_OH was used as solvent to replace CH_3_CN, **3aa** was formed in 98% GC yield (entry 3). In the case of acrylonitrile employed, the yield of the corresponding product **3af** is also greatly depending on the solvents used, and CH_3_OH is the best choice (entries 4–6). Increasing the amount of NaOAc (from 1.0 equiv. to 2.0 equiv.) afforded **3af** in the same yield (entry 7), but the decreasing amount of NaOAc (from 1.0 equiv. to 0.5 equiv.) resulted in the considerable decrease of the yield of **3af** (entry 8). Moreover, the use of Na_2_CO_3_ as base led to 44% of **3af** (entry 9), and the use of 1,4-diazabicyclo[2.2.2]octane (DABCO), 1,8-diazabicyclo[5.4.0]-undec-7-ene (DBU) and pyridine as base, or without the use of base led to no formation of **3af** at all (entries 10–13).

Under the optimized reaction conditions shown in entries 3 and 6 in Table 1, the generality for the formation of alkyl aryl sulfones was investigated by using a variety of *N*-arylsulfonyl hydroxylamines and alkenes, and the obtained results are summarized in Table 2 and Table 3. As shown in Table 2, **1a** reacted with acrylates **2b**–**e** to give the expected alkyl aryl sulfones **3ab**–**3ae** in high yields. No significant electron effect was observed, and when R are both electron-donating and electron-withdrawing groups, *N*-arylsulfonyl hydroxylamines underwent the present reaction smoothly to afford the corresponding alkyl aryl sulfones **3ba**, **3bb**, **3ca**, and **3da**–**fa** in good yields.

In addition, it is also interesting to prepare the multi-sulfur atom-bearing compounds, thus we performed the reactions of **1a** and **1c** with phenyl vinyl sulfone (**2g**). As shown in Table 3, the desired products **3ag** and **3cg** were isolated in good yields.

**Scheme 2:**
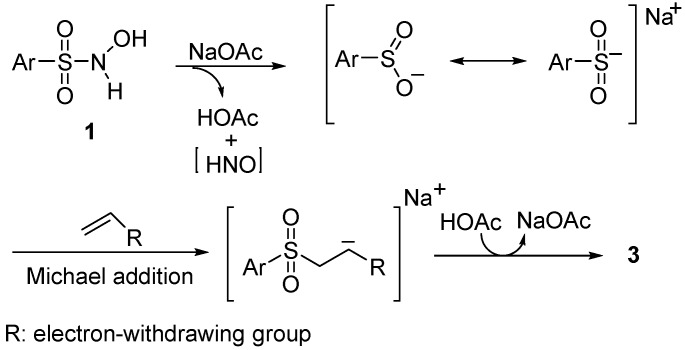
The proposed mechanism for the formation of alkyl aryl sulfones.

On the basis of the known decomposition of *N*-arylsulfonyl hydroxyamines under basic conditions to nitrosyl hydride and arylsulfinate [18], a proposed mechanism for the formation of alkyl aryl sulfones is shown in Scheme 2. It involves the formation of an arylsulfinate anion/arylsulfonyl anion intermediate [12], and its Michael addition with an electron-deficient alkene to afford alkyl aryl sulfone **3**.

## 3. Materials and Methods

### 3.1. General Methods

All organic starting materials and solvents are analytically pure and used without further purification. Nuclear magnetic resonance (NMR) spectra were recorded on a JEOL ECA-300 spectrometer (JEOL, Tokyo, Japan) using CDCl_3_ as a solvent at 298 K. ^1^H-NMR (300 MHz) chemical shifts (δ) were referenced to internal standard TMS (for ^1^H, δ = 0.00 ppm). ^13^C-NMR (75 MHz) chemical shifts were referenced to internal solvent CDCl_3_ (for ^13^C, δ = 77.16 ppm). The ^1^H- and ^13^C-NMR charts of products are reported as supplementary materials. The high-resolution mass spectra (ESI) were obtained with a micrOTOF-Q 10142 spectrometer (Agilent, California, CA, USA).

### 3.2. Typical Experiment Procedure for the Reaction of N-Phenylsulfonyl Hydroxylamine *(**1a**)* with Methyl Acrylate *(**2a**)* Affording Methyl 3-(Phenylsulfonyl)propanoate *(**3aa**)* (Table 1, Entry 3)

A mixture of *N*-phenylsulfonyl hydroxylamine (**1a**, 173.0 mg, 1.0 mmol), methyl acrylate (**2a**) (172.0 mg, 2.0 mmol) and NaOAc (82.0 mg, 1.0 mmol) was heated in CH_3_OH (10.0 mL) at 60 °C (oil bath temperature) with stirring for 18 h in a screw-capped thick-walled Pyrex tube under an air atmosphere. After the reaction mixture was cooled to room temperature, it was directly subjected to a short silica column chromatography (2~3 cm, eluted with CH_2_Cl_2_) to remove the insoluble materials. To the collected solution, *n*-octadecane (25.5 mg, 0.1 mmol as internal standard for GC analysis) was then added with stirring. After GC and GC-MS analyses of the mixture, volatiles were then removed under reduced pressure, and the residue was subjected to silica gel column chromatography, eluted with a mixture of solvents of petroleum ether/acetone (from 100:0~100:2 in volume). **3aa** was obtained in 212.0 mg (0.93 mmol, 93%) as yellow oil. The GC analysis of reaction mixture revealed the formation of **3aa** [19] in 98% GC yield. ^1^H-NMR (CDCl_3_) δ 7.85 (m, 2H), 7.56 (m, 3H), 3.56 (s, 3H), 3.39 (t, 2H, *J* = 7.8 Hz), 2.70 (t, 2H, *J* = 7.8 Hz); ^13^C-NMR (CDCl_3_) δ 170.3, 138.6, 134.1, 129.4, 128.1, 52.2, 51.4, 27.6; HRMS (ESI): Calcd. for: C_10_H_13_O_4_S [M + H]^+^: 229.0529; found: 229.0528.

The following compounds were similar prepared:

*Ethyl 3-(phenylsulfonyl)propanoate* (**3ab**) [20]: ^1^H-NMR: δ 7.80 (m, 2H), 7.57 (m, 3H), 4.04 (dd, 2H, *J* = 7.2 Hz), 3.40 (t, 2H, *J* = 7.8 Hz), 2.70 (t, 2H, *J* = 7.8 Hz),1.18 (t, 3H, *J* = 7.2 Hz); ^13^C-NMR: δ 170.0, 138.4, 134.0, 129.4, 128.1,61.3, 51.4, 27.8, 14.0; HRMS (ESI): Calcd. for: C_11_H_12_NO_4_S [M + H]^+^: 254.0482; found: 254.0486.

*n-Butyl 3-(phenylsulfonyl)propanoate* (**3ac**): pale yellow oil; ^1^H-NMR: δ 7.89 (m, 2H), 7.63 (m, 3H), 4.03 (m, 2H), 3.45 (t, 2H, *J* = 7.5 Hz), 2.73 (t, 2H, *J* = 7.5 Hz), 1.56 (m, 2H), 1.33 (m, 2H), 0.90 (m, 3H); ^13^C-NMR: δ 169.7, 138.3, 133.8, 129.2, 127.9, 64.9, 51.2, 30.2, 27.6, 18.8, 13.4; HRMS (ESI): Calcd. for: C_13_H_19_O_4_S [M + H]^+^: 271.0999; found: 271.0998.

*t-Butyl 3-(phenylsulfonyl)propanoate* (**3ad**): yellow solid, m.p. 46~48 °C; ^1^H-NMR: δ 7.91 (m, 2H),7.62 (m, 3H), 3.37 (t, 2H, *J* = 7.8 Hz), 2.65 (t, 2H, *J* = 7.8 Hz), 1.38 (s, 9H); ^13^C-NMR: δ 169.2, 138.7, 134.0, 129.5, 128.2, 82.0, 51.7, 29.0, 28.1; HRMS (ESI): Calcd. for: C_13_H_19_O_4_S [M + Na]^+^: 293.0818; found: 293.0819.

*Benzyl 3-(phenylsulfonyl)propanoate* (**3ae**): pale yellow solid, m.p. 61~63 °C; ^1^H-NMR: δ 7.89 (m, 2H), 7.59 (m, 3H), 7.32 (m, 5H), 5.05 (s, 2H), 3.43 (t, 2H, *J* = 7.8 Hz), 2.73 (t, 2H, *J* = 7.8 Hz); ^13^C-NMR: δ 169.7, 138.3, 135.1, 134.0, 139.4, 128.6, 128.4, 128.3, 128.1, 67.0, 51.3, 27.8; HRMS (ESI): Calcd. for: C_16_H_16_NaO_4_S [M + Na]^+^: 327.0662; found: 327.0666.

*3-(Phenylsulfonyl)propanenitrile* (**3af**) [19]: ^1^H-NMR: δ 7.94 (m, 2H), 7.69 (m, 3H), 7.32 (m, 5H), 3.40 (t, 2H, *J* = 7.6 Hz), 2.82 (t, 2H, *J* = 7.6 Hz); ^13^C-NMR: δ 137.5, 134.8, 129.8, 128.3, 116.1, 51.1, 12.1; HRMS (ESI): Calcd. for: C_9_H_9_NNaO_2_S [M + Na]^+^: 218.0246; found: 218.0249.

*Methyl 3-tosylpropanoate* (**3ba**) [20]: ^1^H-NMR: δ 7.72 (d, 2H, *J* = 8.2 Hz), 7.31 (d, 2H, *J* = 8.2 Hz), 3.57 (s, 3H), 3.35 (t, 2H, *J* = 7.5 Hz), 2.67 (t, 2H, *J* = 7.5 Hz), 2.39 (s, 3H); ^13^C-NMR: δ 170.4, 145, 135.4, 129.9, 128.1, 52.1, 51.4, 27.6, 21.5; HRMS (ESI): Calcd. for: C_11_H_15_O_4_S [M + H]^+^: 243.0686; found: 243.0690.

*Ethyl 3-tosylpropanoate* (**3bb**) [20]: ^1^H-NMR: δ 7.74 (d, 2H, *J* = 7.8 Hz), 7.32 (d, 2H, *J* = 7.8 Hz), 4.03 (q, 2H, *J* = 7.1 Hz), 3.36 (t, 2H, *J* = 7.6 Hz), 2.66 (t, 2H, *J* = 7.6 Hz), 2.40 (s, 3H), 1.17 (t, 2H, *J* = 7.1 Hz), 0.90 (m, 3H); ^13^C-NMR: δ 170.0, 145.0, 135.5, 130.0, 128.1, 61.3, 51.5, 27.9, 21.6, 14.0; HRMS (ESI): Calcd. for: C_12_H_17_O_4_S [M + H]^+^: 257.0842; found: 257.0844.

*Methyl 3-((4-methoxyphenyl)sulfonyl)propanoate* (**3ca**) [20]: ^1^H-NMR: δ 7.83 (d, 2H, *J* = 9.0 Hz), 7.04 (d, 2H, *J* = 9.0 Hz), 3.89 (s, 3H), 3.64 (s, 3H), 3.41 (t, 2H, *J* = 7.1 Hz), 2.74 (t, 2H, *J* = 7.1 Hz); ^13^C-NMR: δ 170.4, 163.9, 130.3, 129.8, 114.5, 55.7, 52.2, 51.6, 27.7; HRMS (ESI): Calcd. for: C_11_H_15_O_5_S [M + H]^+^: 259.0635; found: 259.0637.

*Methyl 3-((4-chlorophenyl)sulfonyl)propanoate* (**3da**) [21]: ^1^H-NMR: δ 7.78 (d, 2H, *J* = 8.5 Hz), 7.49 (d, 2H, *J* = 8.5 Hz), 3.55 (s, 3H), 3.38 (t, 2H, *J* = 7.6 Hz), 2.67 (t, 2H, *J* = 7.6 Hz); ^13^C-NMR: δ 170.1, 140.5, 136.8, 129.6, 52.2, 51.3, 27.4; HRMS (ESI): Calcd. for: C_10_H_11_ClNaO_4_S [M + Na]^+^: 284.9959; found: 284.9962.

*Methyl 3-((4-(trifluoromethyl)phenyl)sulfonyl)propanoate* (**3ea**): white solid, m.p. 120~122 °C; ^1^H-NMR: δ 8.06 (d, 2H, *J* = 8.2 Hz), 7.85 (d, 2H, *J* = 8.6 Hz), 3.64 (s, 3H), 3.47 (t, 2H, *J* = 7.5 Hz), 2.77 (t, 2H, *J* = 7.5 Hz); ^13^C-NMR: δ 170.2, 142.0, 135.7, 128.9, 127.0, 126.6, 52.4, 51.4, 27.4; HRMS (ESI): Calcd. for: C_11_H_11_F_3_NaO_4_S [M + Na]^+^: 319.0222; found: 319.0226.

*Methyl 3-((4-cyanophenyl)sulfonyl)propanoate* (**3fa**): pale yellow solid, m.p. 119~122 °C; ^1^H-NMR: δ 8.03 (m, 2H), 7.87 (m, 2H), 3.64 (s, 3H), 3.46 (t, 2H, *J* = 7.5 Hz), 2.77 (t, 2H, *J* = 7.5 Hz); ^13^C-NMR: δ 17.02, 142.8, 133.3, 129.1, 118.0, 117.1, 52.6, 51.5, 27.4; HRMS (ESI): Calcd. for: C_11_H_12_NO_4_S [M + H]^+^: 254.0482; found: 254.0486.

*1,2-Bis(phenylsulfonyl)ethane* (**3ag**) [22]: ^1^H-NMR: δ 7.88 (m, 4H), 7.70 (m, 2H), 7.60 (m, 6H), 3.45 (s, 4H); ^13^C-NMR: δ 138.0, 134.6, 129.7, 128.1, 49.5.

*1-Methoxy-4-((2-(phenylsulfonyl)ethyl)sulfonyl)benzene* (**3cg**): white solid, m.p. 150~152 °C; ^1^H-NMR: δ 7.87 (d, 2H, *J* = 7.6 Hz), 7.78 (d, 2H, *J* = 8.8 Hz, 7.70 (t, 1H, *J* = 7.4 Hz ), 7.58 (t, 2H, *J* = 7.7 Hz ), 3.88 (s, 3H), 3.42 (m, 4H); ^13^C-NMR: δ 164.4, 138.1, 134.6, 130.4, 129.8, 129.4, 128.1, 114.5, 55.9, 49.8; HRMS (ESI): Calcd. for: C_15_H_17_O_5_S_2_ [M + H]^+^: 341.0512; found: 341.0515.

## 4. Conclusions

In summary, we have studied the reaction of *N*-arylsulfonyl hydroxylamines with electron-deficient alkenes to provide an alternative efficient synthetic method for the formation of alkyl aryl sulfones in good yields. The present method has the advantages with simple and easily available starting materials, under mild conditions, with high atom-utilization.

## Figures and Tables

**Table 1:**
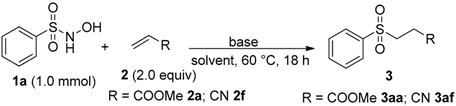
Optimizing the reaction conditions ^a^.

Entry	R	Solvent	Base (Equiv.)	Yield (%) ^b^
1	COOMe	CH_3_CN	--	0
2		CH_3_CN	NaOAc (1)	39
3		CH_3_OH	NaOAc (1)	98(93)
4	CN	toluene	NaOAc (1)	45
5		CH_3_CN	NaOAc (1)	53
6		CH_3_OH	NaOAc (1)	85(80)
7		CH_3_OH	NaOAc (2)	84
8		CH_3_OH	NaOAc (0.5)	67
9		CH_3_OH	Na_2_CO_3_	44
10		CH_3_OH	DABCO (1)	0
11		CH_3_OH	DBU (1)	0
12		CH_3_OH	pyridine	0
13		CH_3_OH	--	0

^a^ Reactions were carried out using 1.0 mmol of **1a**, 2.0 mmol of **2a** or **2f**, and base in 10.0 mL of solvent in a sealed tube. ^b^ GC yield based on the amount of **1a** used. Number in parenthesis is isolated yield.

**Table 2:**
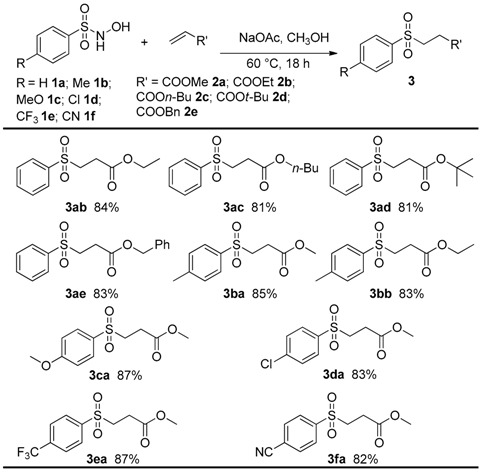
Synthesis of alkyl aryl sulfones ^a^.

^a^ Reactions were carried out using 1.0 mmol of **1**, 2.0 mmol of **2**, and 1.0 mmol of NaOAc in 10.0 mL of MeOH at 60 °C for 18 h. All the yields are isolated yields.

**Table 3:**
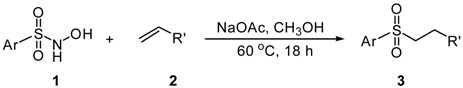
Synthesis of alkyl aryl sulfones ^a^.

Entry	1	2	3	Yield (%) ^b^
1	**1a**	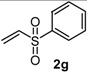	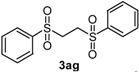	75
2	**1c**	**2g**	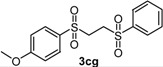	76

^a^ Reactions were carried out using 1.0 mmol of **1**, 2.0 mmol of **2**, and 1.0 mmol of NaOAc in 10.0 mL of MeOH at 60 °C for 18 h. ^b^ Isolated yields.

## Supplementary Materials

Supplementary materials can be accessed at: http://www.mdpi.com/1420-3049/22/1/39/s1, the charts of ^1^H- and ^13^C-NMR of products.
