# Supplementary material for: Synthesis of Alkyl Aryl Sulfones via Reaction of N-Arylsulfonyl Hydroxyamines with Electron-Deficient Alkenes
Source: Molecules. 2016 Dec 28;22(1):39. doi: 10.3390/molecules22010039 (PMC6155654; doi:10.3390/molecules22010039)
Supplement: Supplementary file 1 [file molecules-22-00039-s001.pdf]

# Supplementary Materials: Synthesis of Alkyl Aryl Sulfones via Reaction of *N*-Arylsulfonyl Hydroxyamines with Electron-Deficient Alkenes

**Yunhui Bin and Ruimao Hua**

## $^{1}$H- and $^{13}$C-NMR Charts of Products

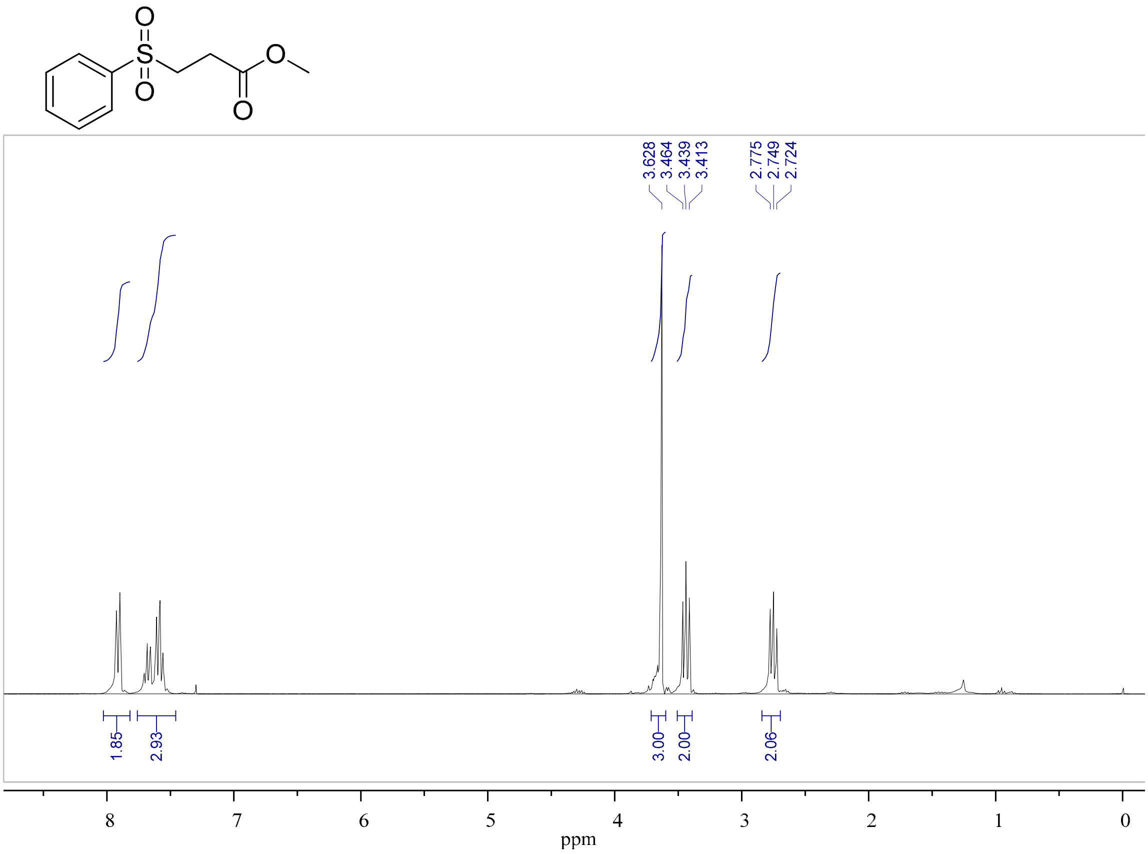


**Figure S1.** $^{1}$H-NMR spectrum of **3aa** (300 MHz, $CDCl_3$).

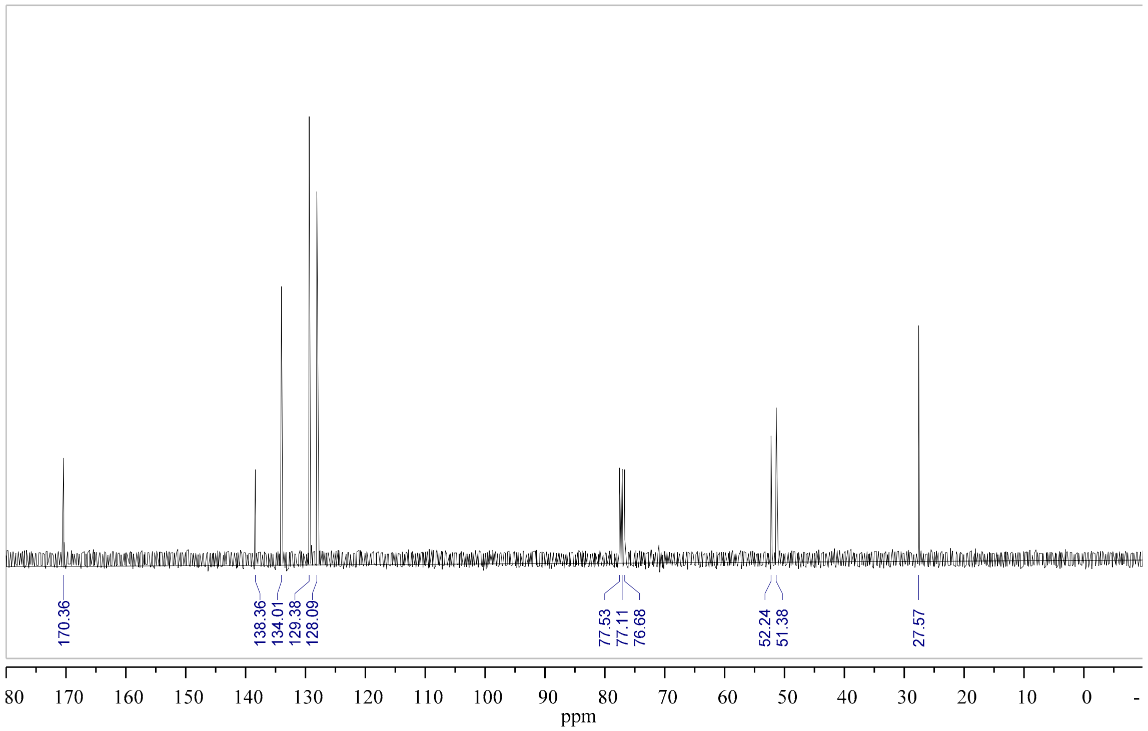


**Figure S2.** $^{13}$C-NMR spectrum of **3aa** (75 MHz, $CDCl_3$).

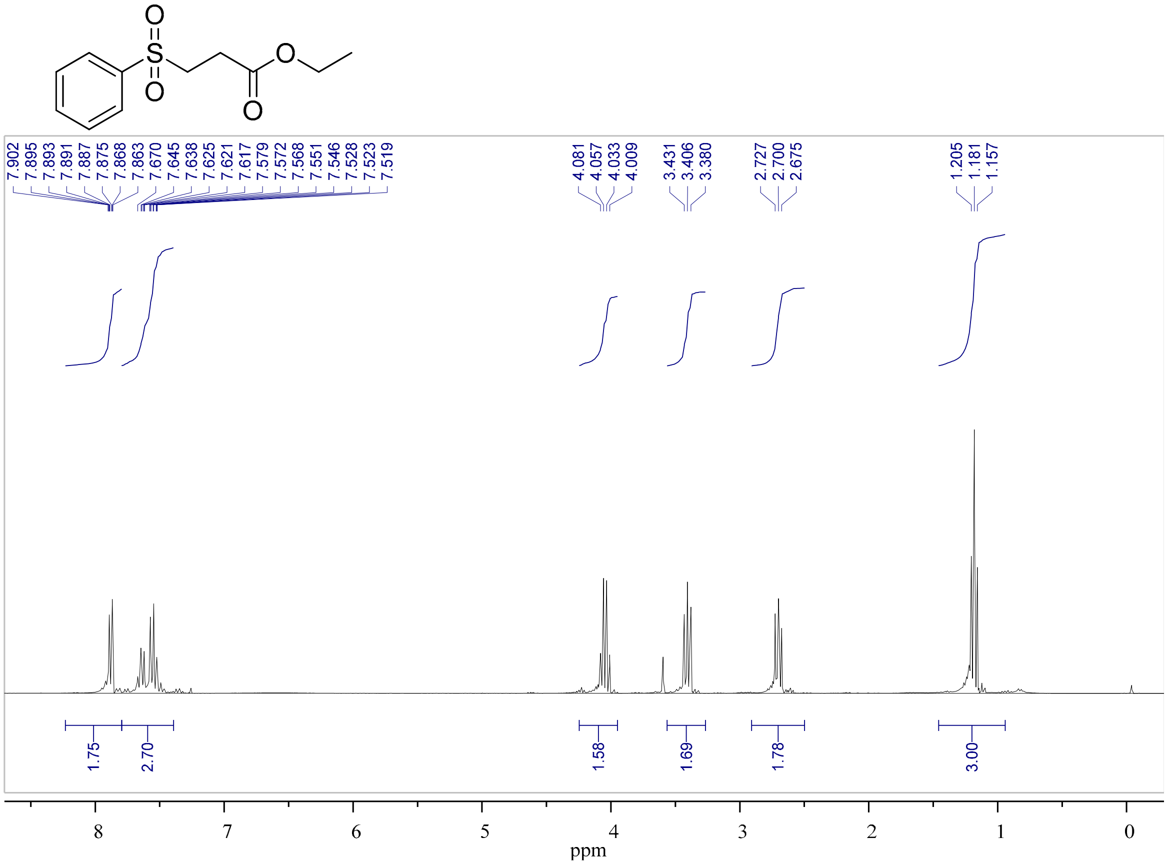


**Figure S3.** $^{1}$H-NMR spectrum of **3ab** (300 MHz, $CDCl_3$).

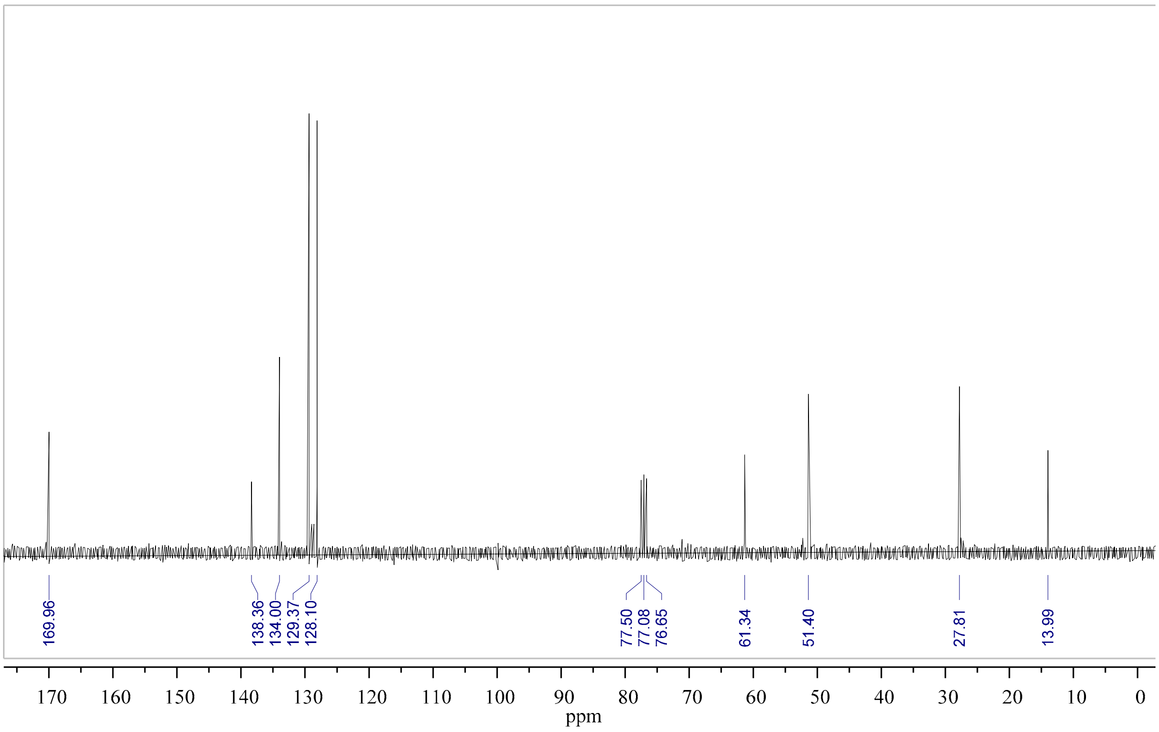


**Figure S4.** $^{13}$C-NMR spectrum of 3ab (75 MHz, $CDCl_3$).

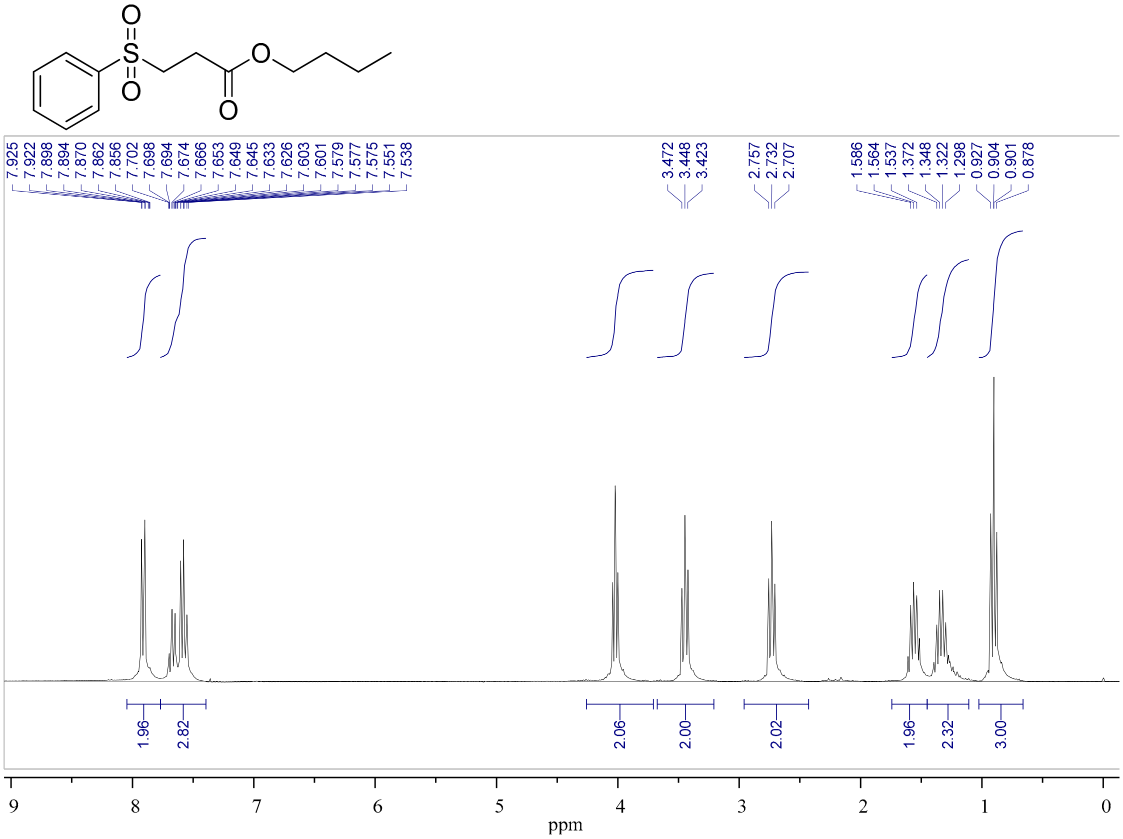


**Figure S5.** $^{1}$H-NMR spectrum of **3ac** (300 MHz, $CDCl_3$).

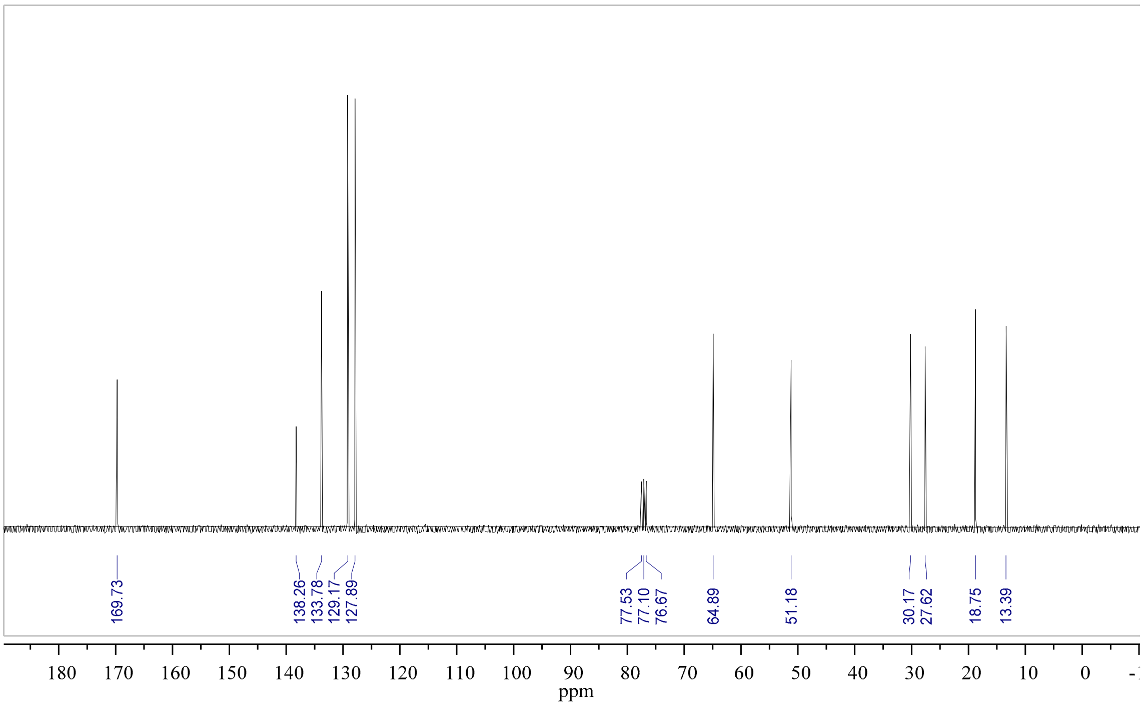


**Figure S6.** $^{13}$C-NMR spectrum of **3ac** (75 MHz, $CDCl_3$).

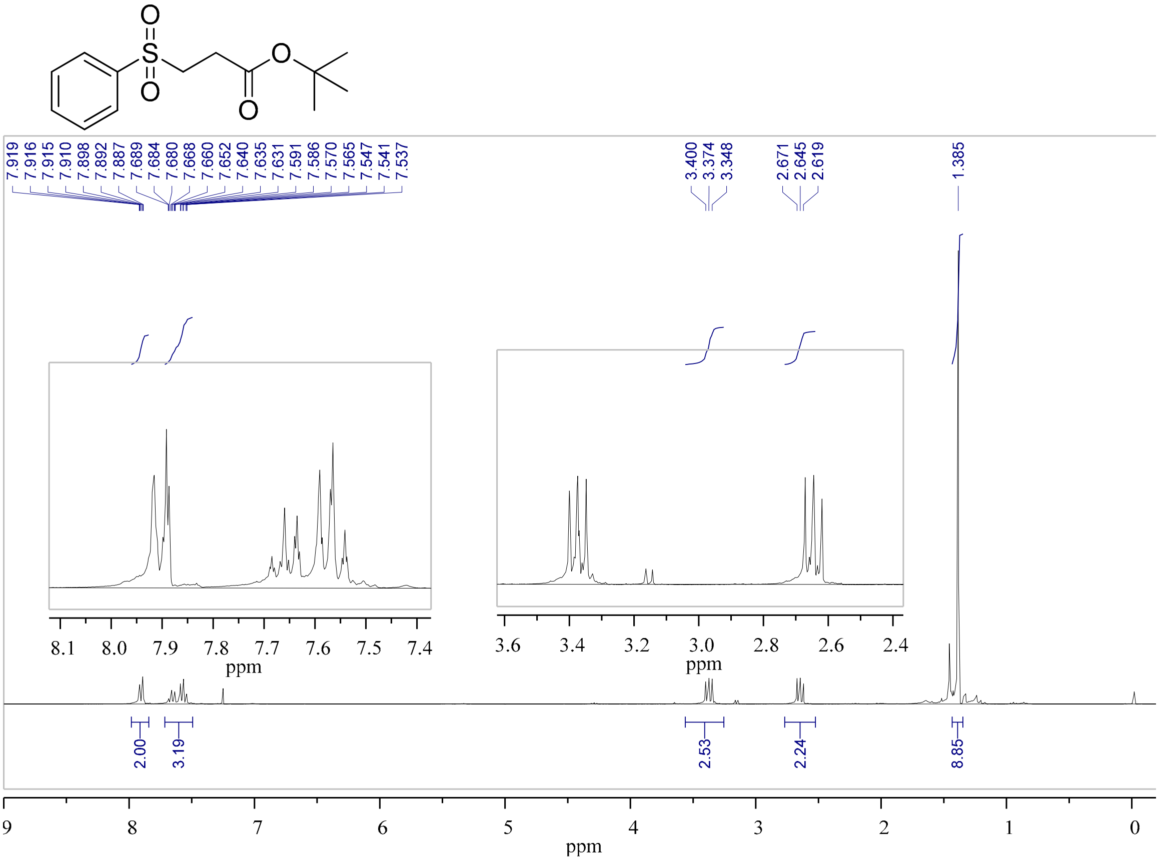


**Figure S7.** $^{1}$H-NMR spectrum of **3ad** (300 MHz, $CDCl_3$).

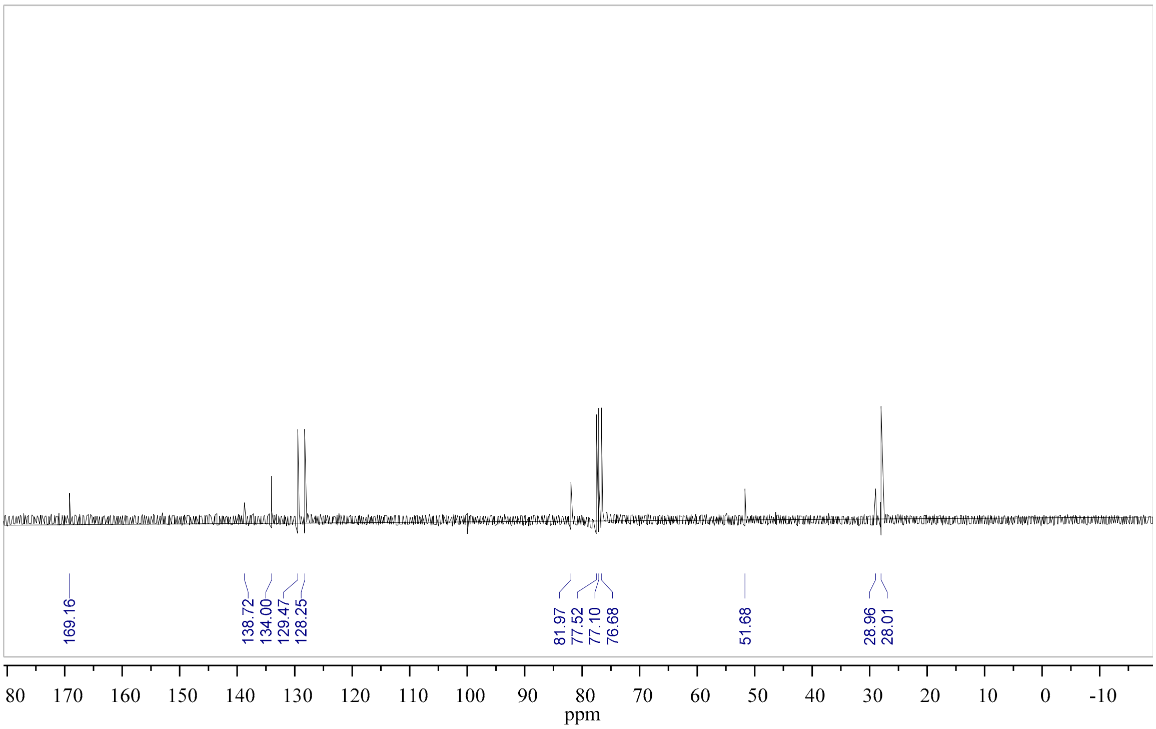


**Figure S8.** $^{13}$C-NMR spectrum of **3ad** (75 MHz, $CDCl_3$).

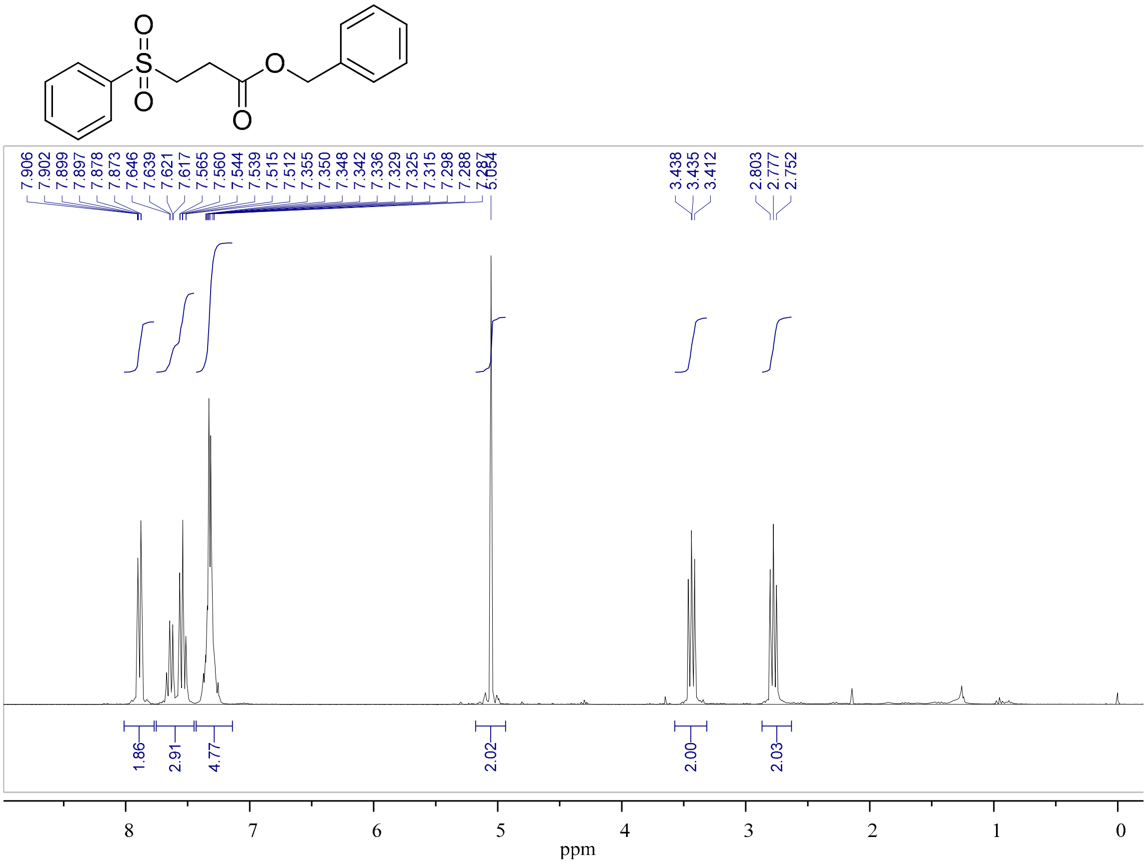


**Figure S9.** $^{1}$H-NMR spectrum of **3ae** (300 MHz, $CDCl_3$).

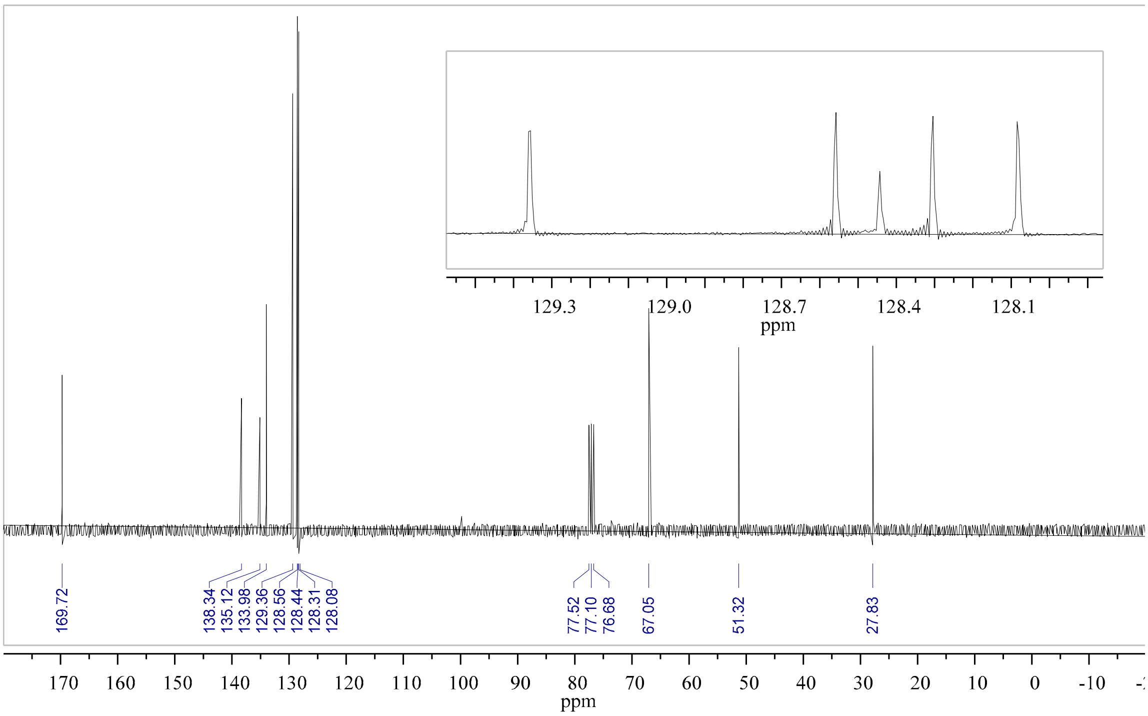


**Figure S10.** $^{13}$C-NMR spectrum of **3ae** (75 MHz, $CDCl_3$).

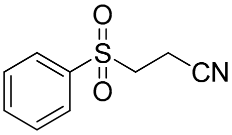


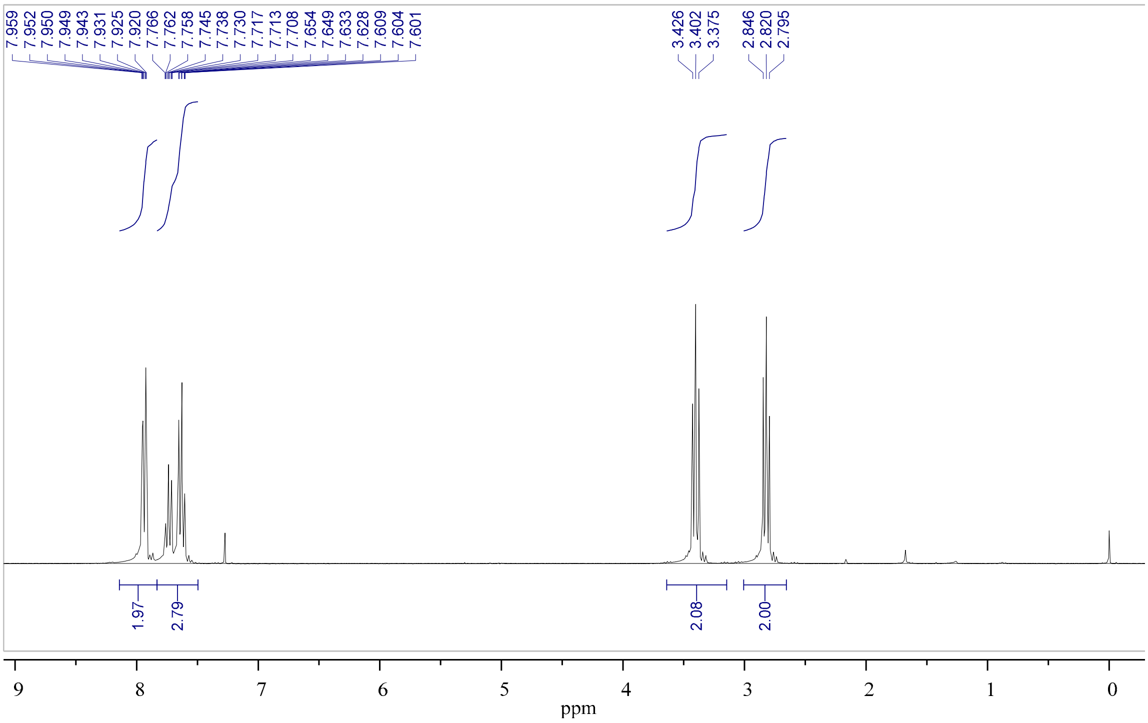


**Figure S11.** $^{1}$H-NMR spectrum of **3af** (300 MHz, $CDCl_3$).

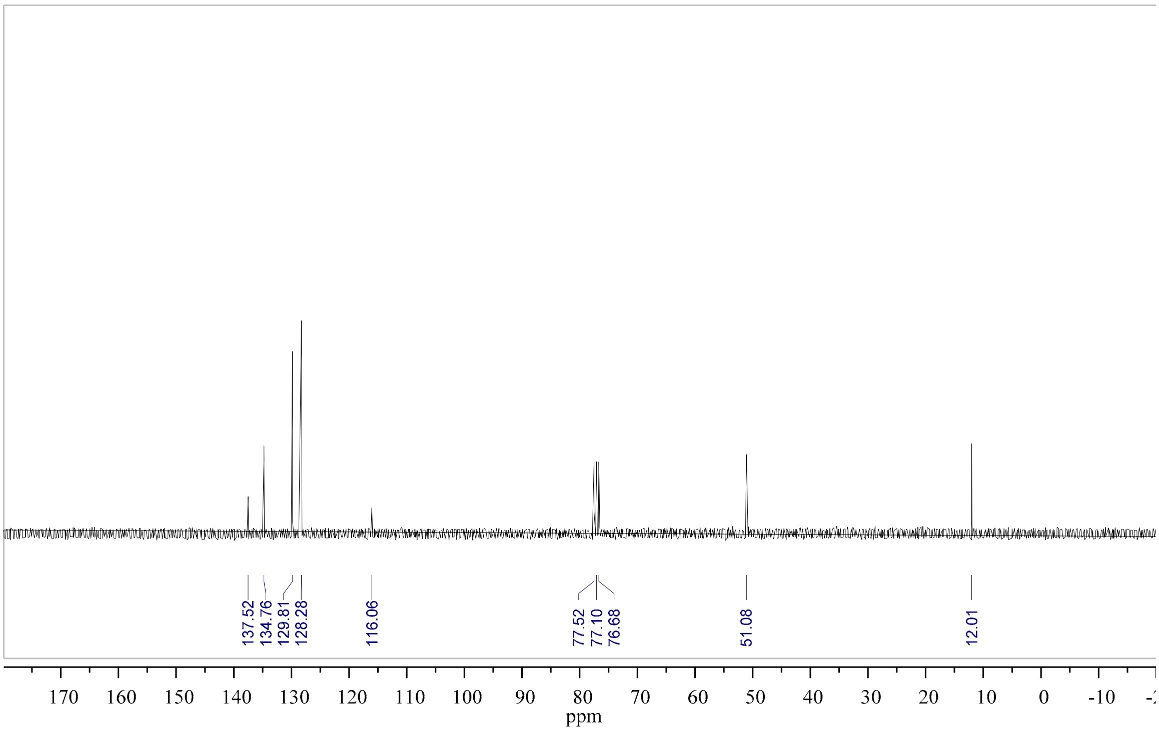


**Figure S12.** $^{13}$C-NMR spectrum of **3af** (75 MHz, $CDCl_3$).

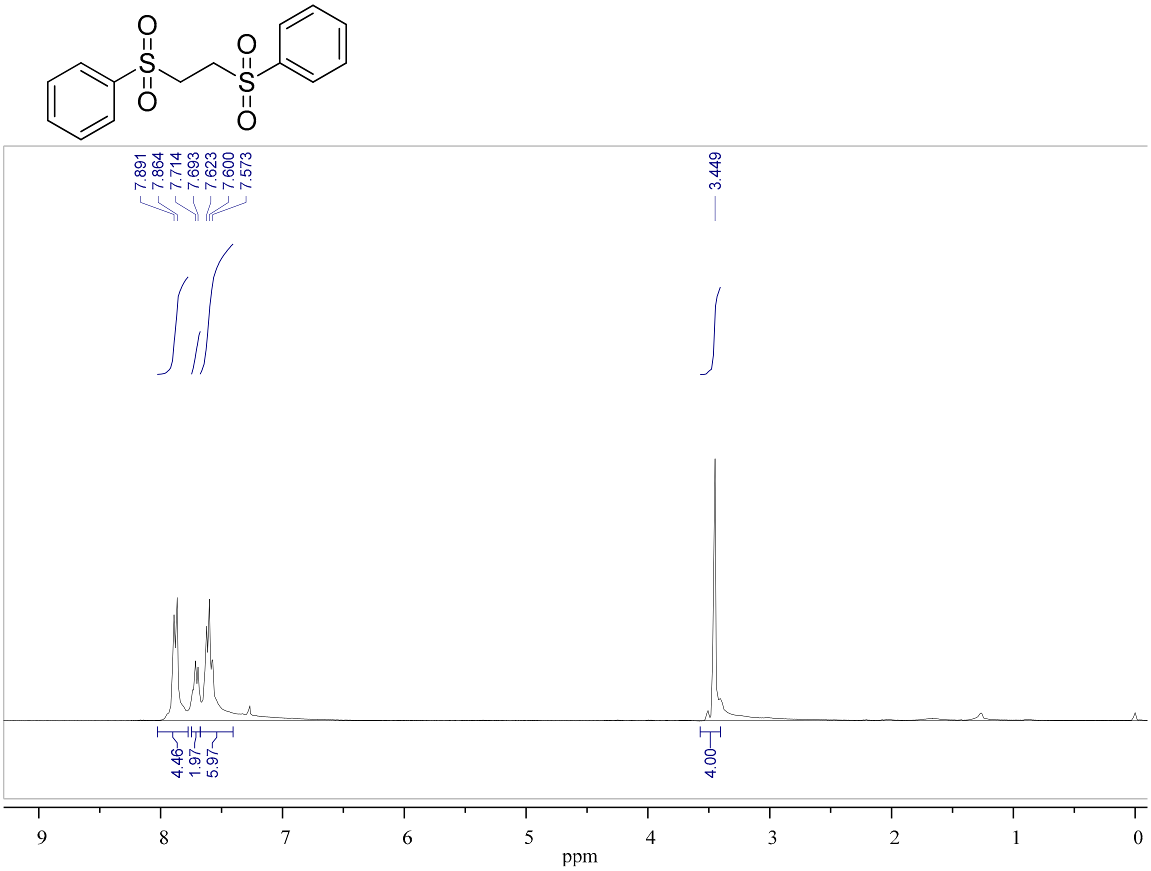


**Figure S13.** $^{1}$H-NMR spectrum of **3ag** (300 MHz, $CDCl_3$).

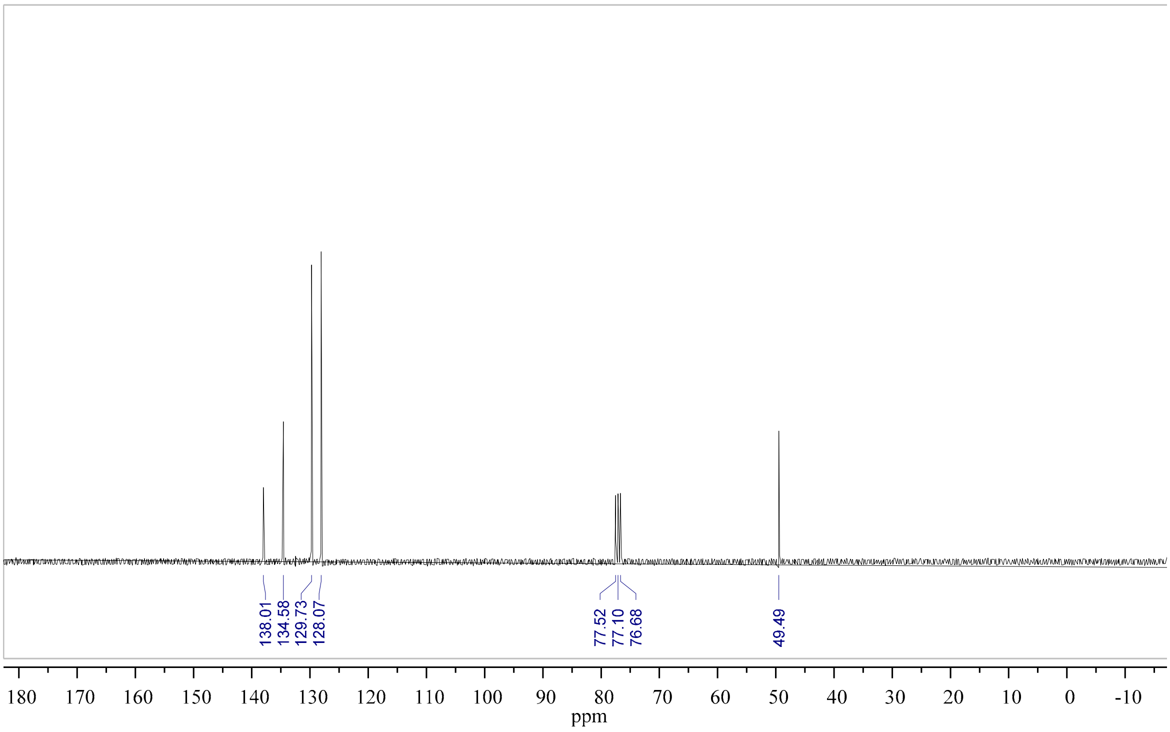


**Figure S14.** $^{13}$C-NMR spectrum of **3ag** (75 MHz, $CDCl_3$).

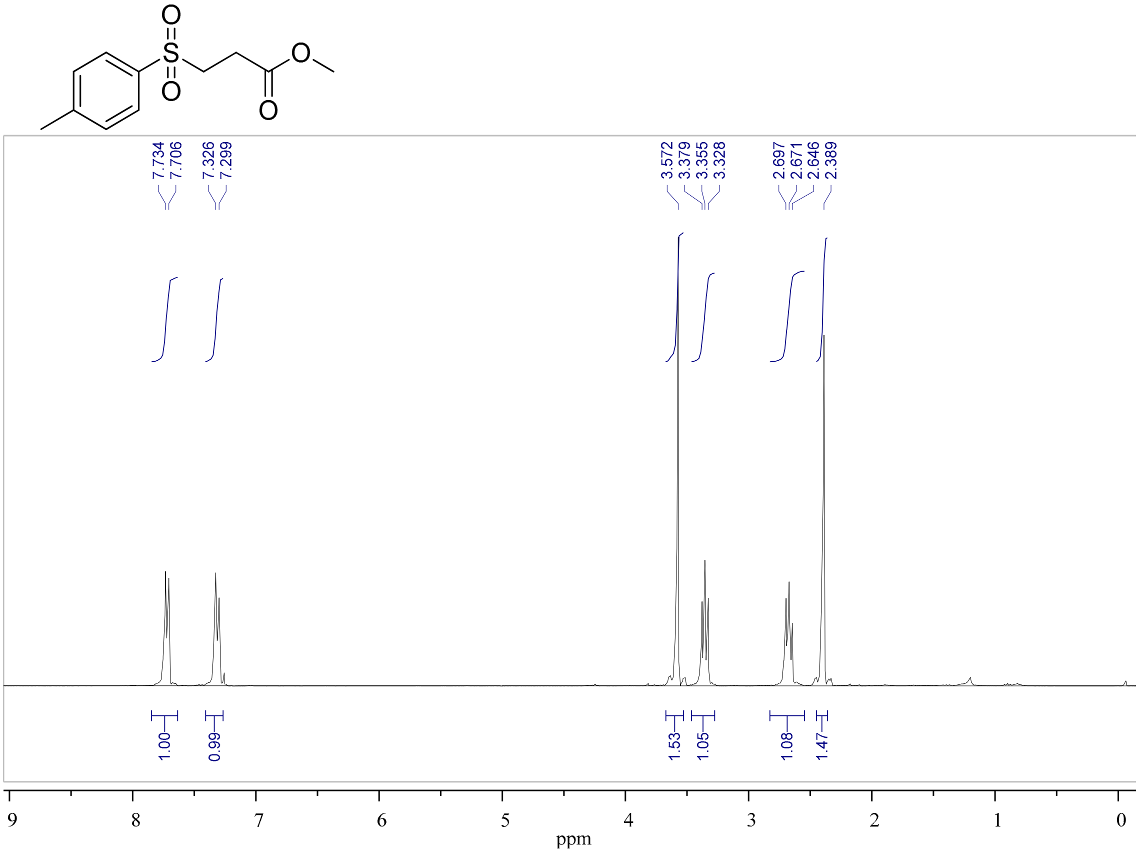


**Figure S15.** $^{1}$H-NMR spectrum of **3ba** (300 MHz, $CDCl_3$).

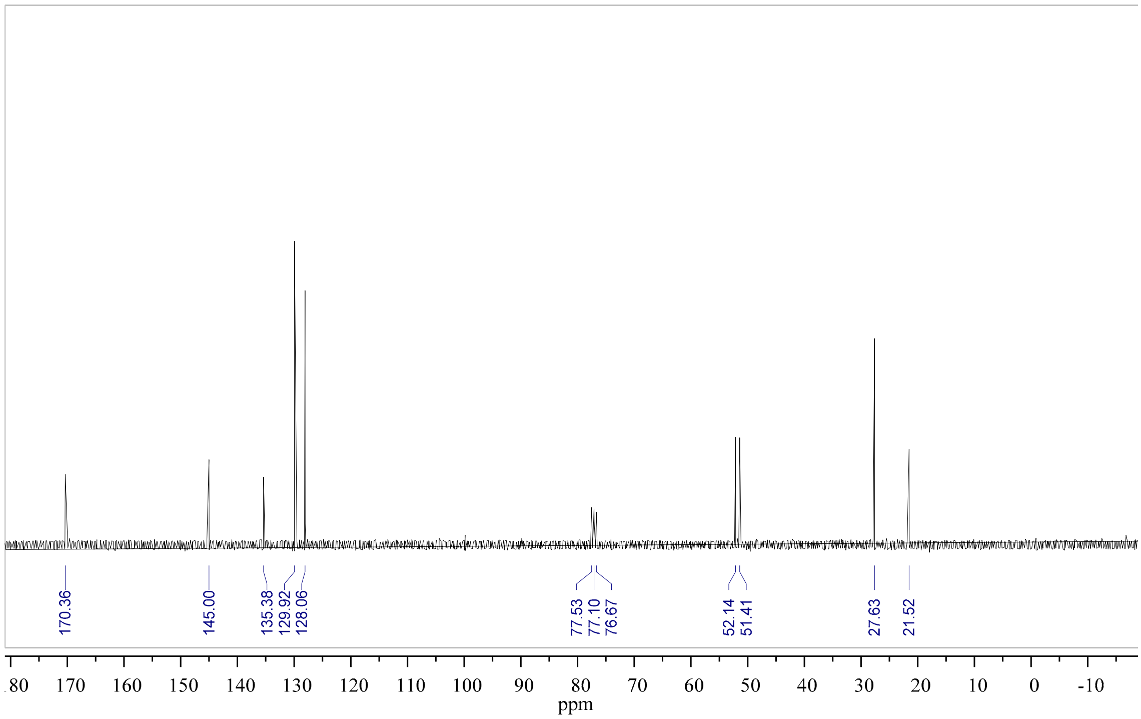


**Figure S16.** $^{13}$C-NMR spectrum of **3ba** (75 MHz, $CDCl_3$).

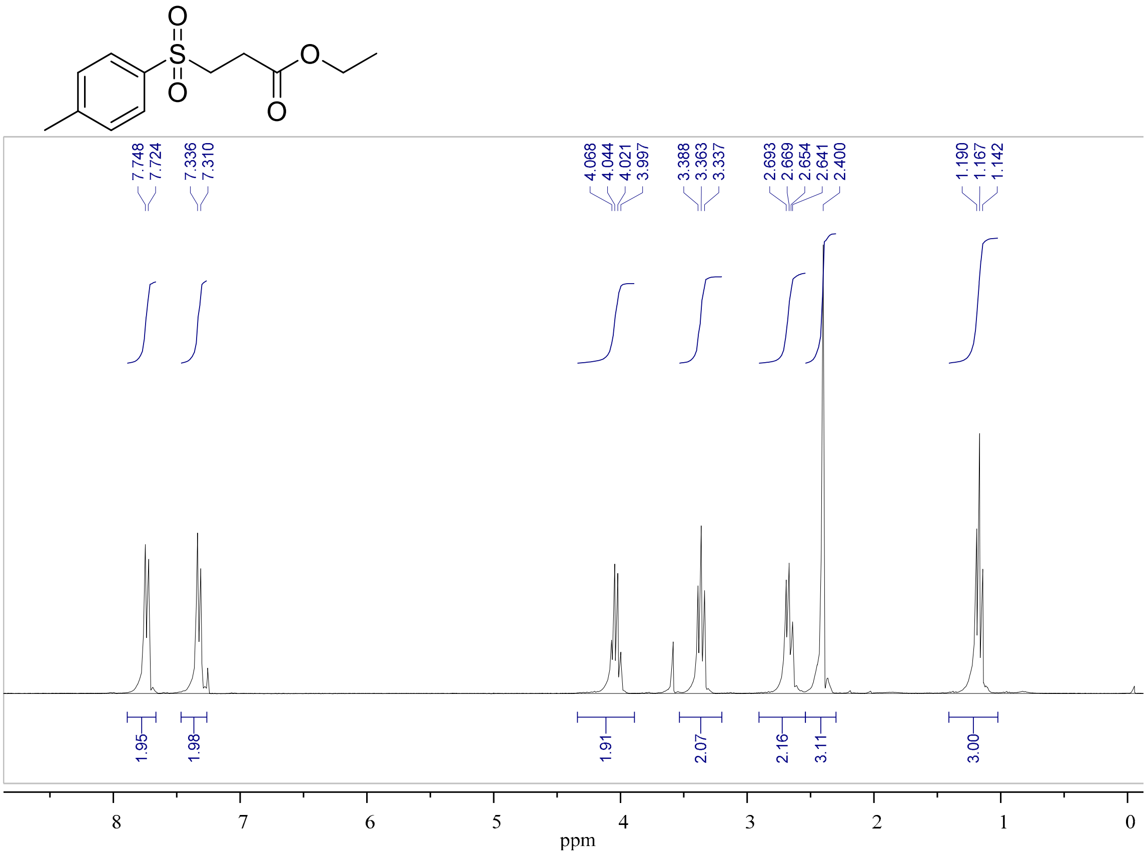


**Figure S17.** $^{1}$H-NMR spectrum of **3bb** (300 MHz, $CDCl_3$).

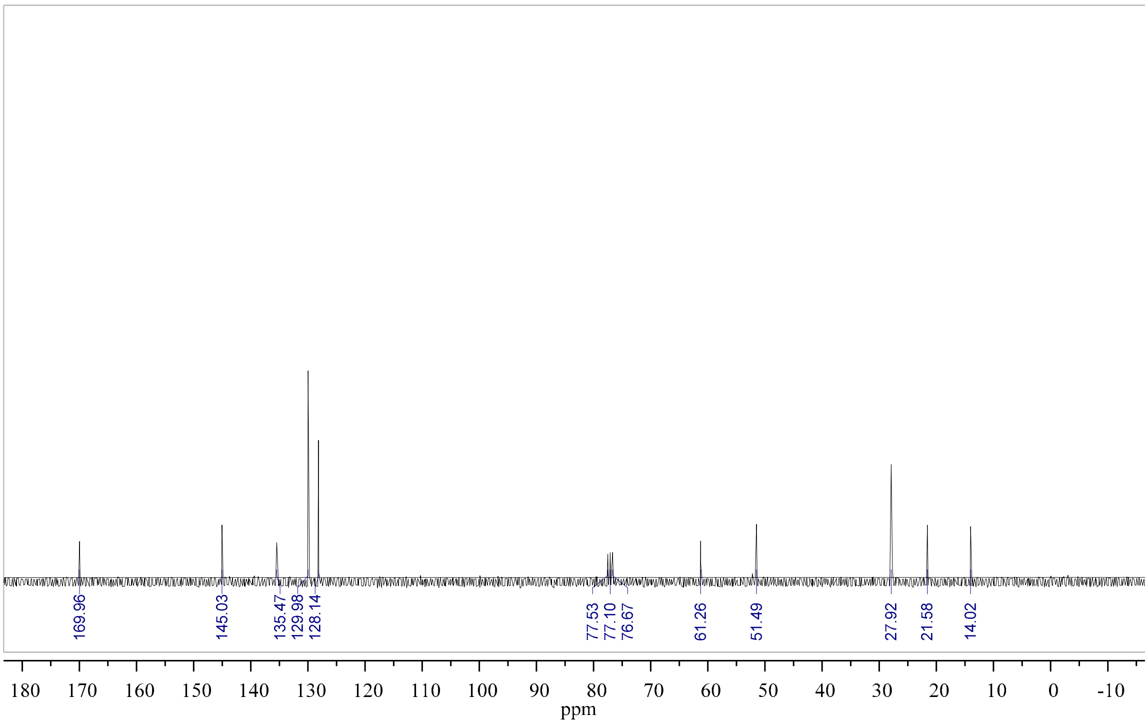


**Figure S18.** $^{13}$C-NMR spectrum of **3bb** (75 MHz, $CDCl_3$).

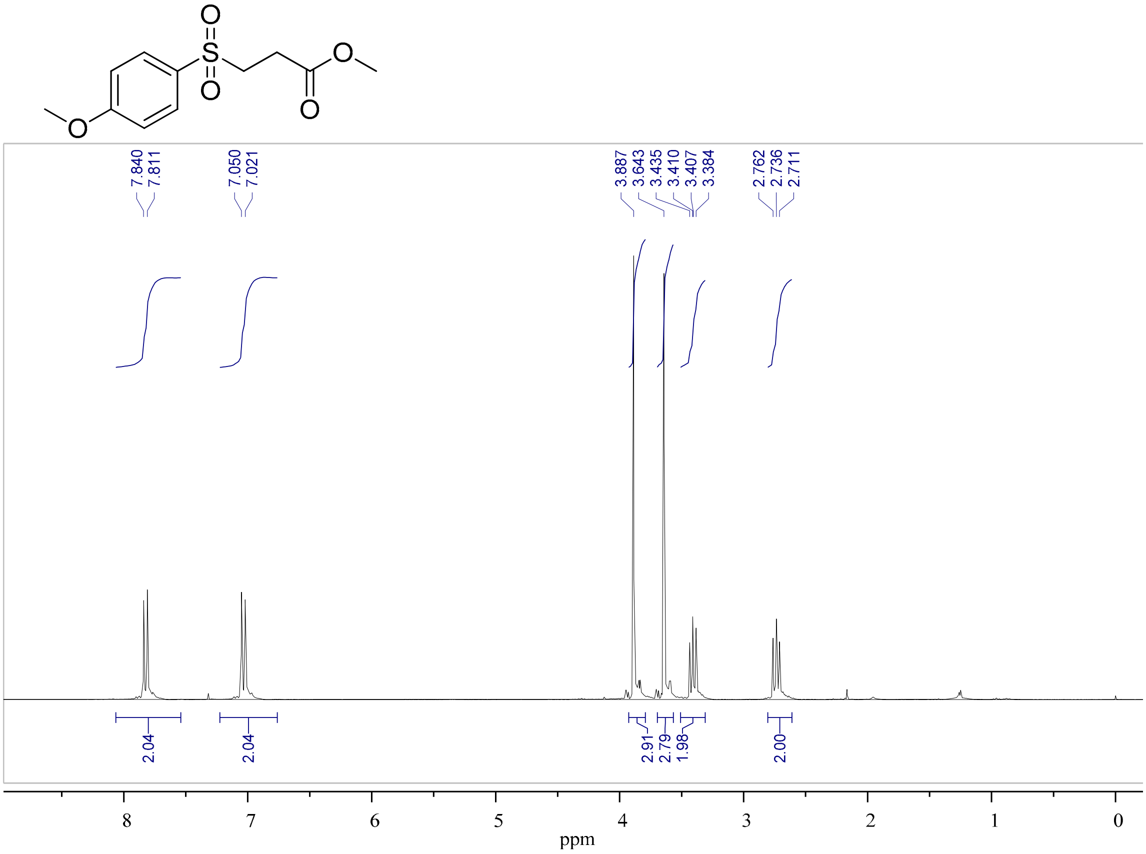


**Figure S19.** $^{1}$H-NMR spectrum of **3ca** (300 MHz, $CDCl_3$).

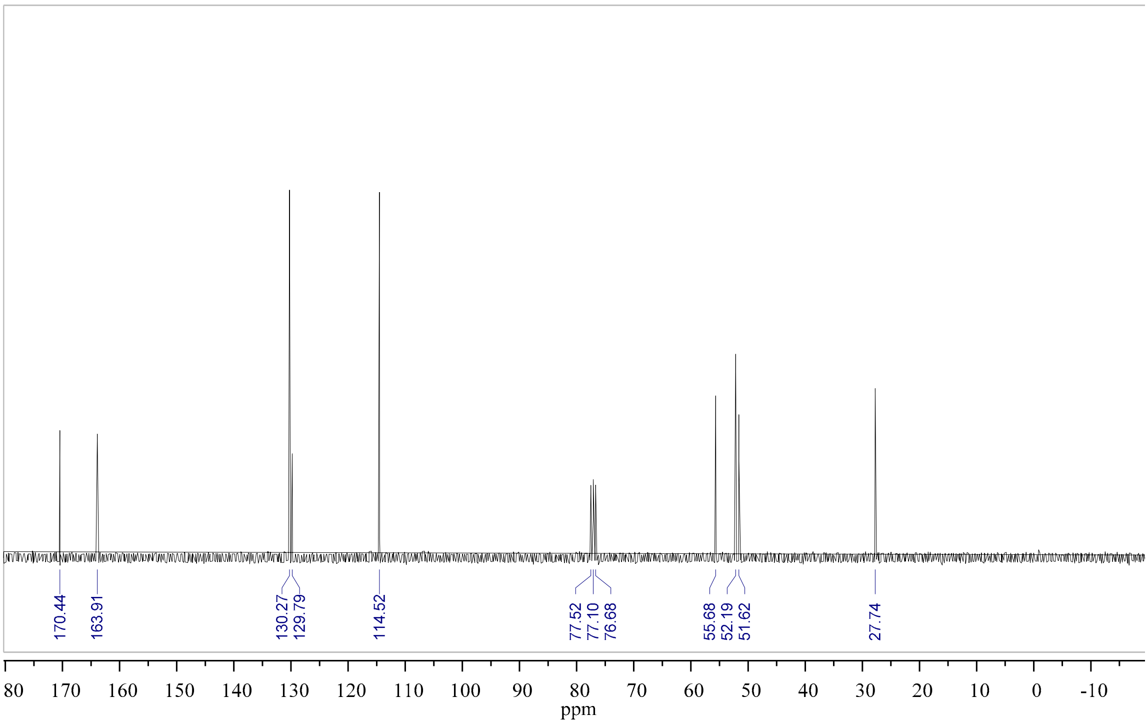


**Figure S20.** $^{13}$C-NMR spectrum of **3ca** (75 MHz, $CDCl_3$).

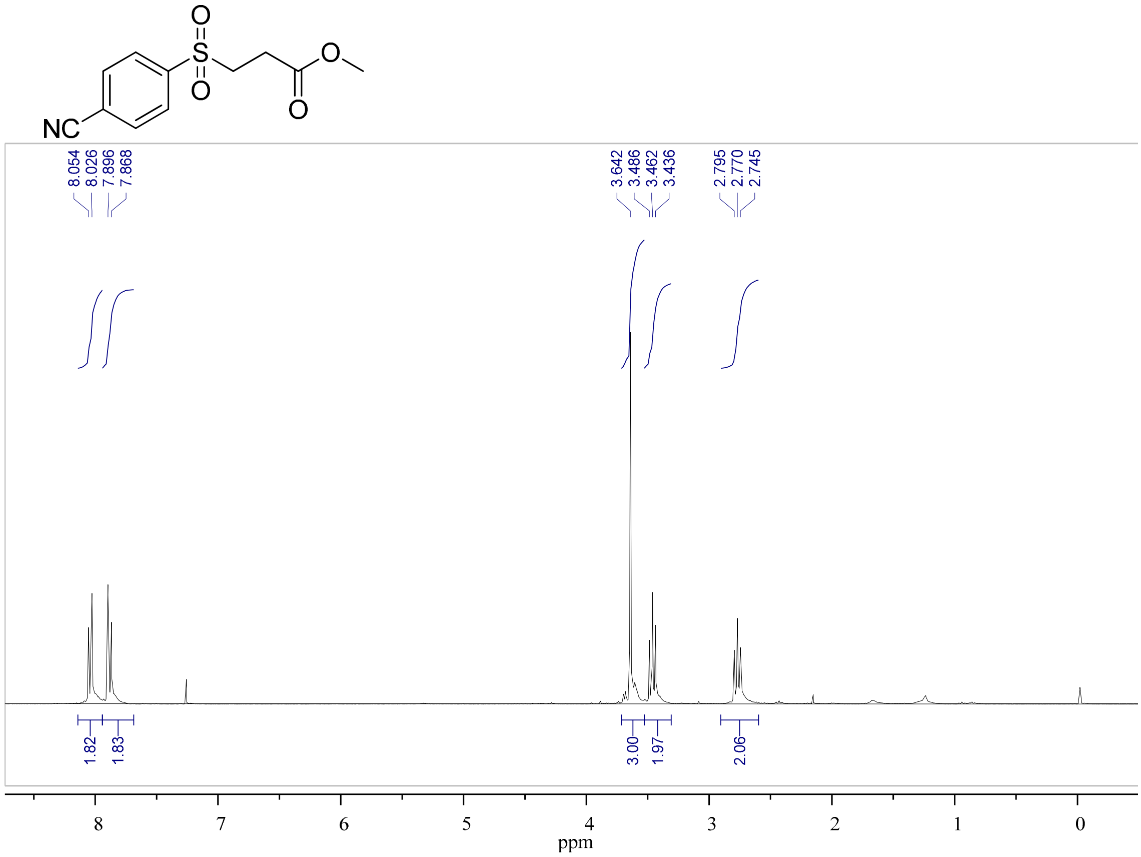


**Figure S21.** $^{1}$H-NMR spectrum of **3fa** (300 MHz, $CDCl_3$).

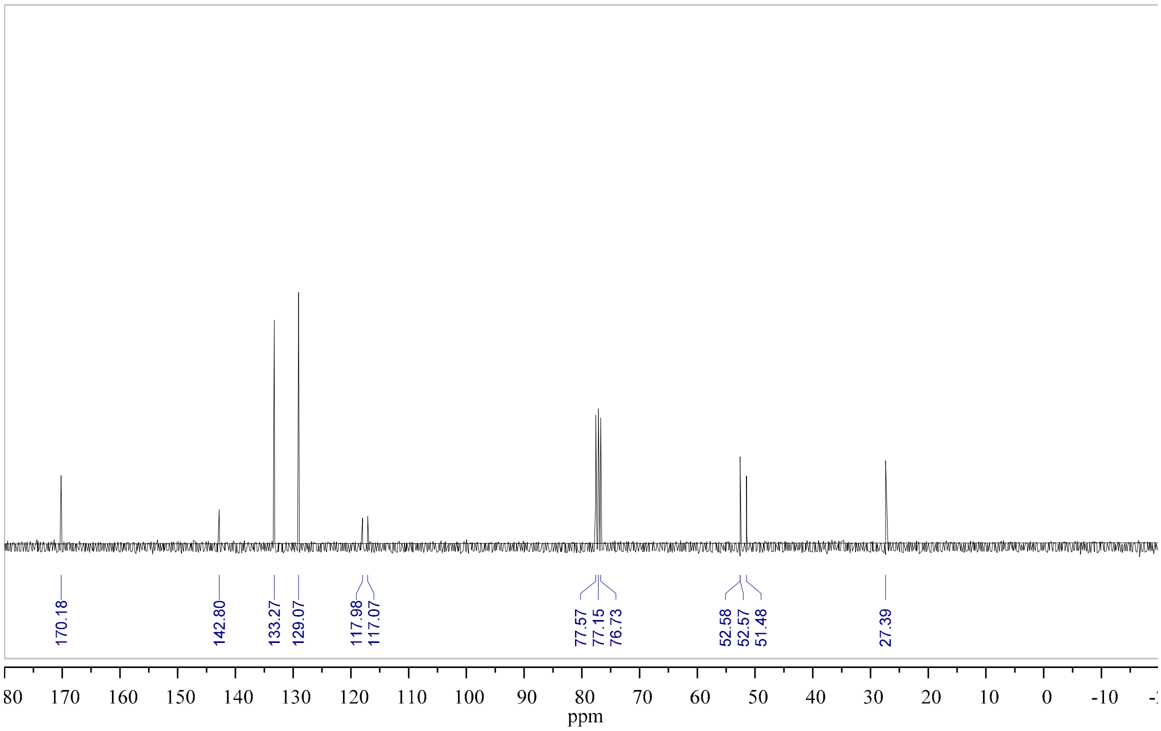


**Figure S22.** $^{13}$C-NMR spectrum of **3fa** (75 MHz, $CDCl_3$).

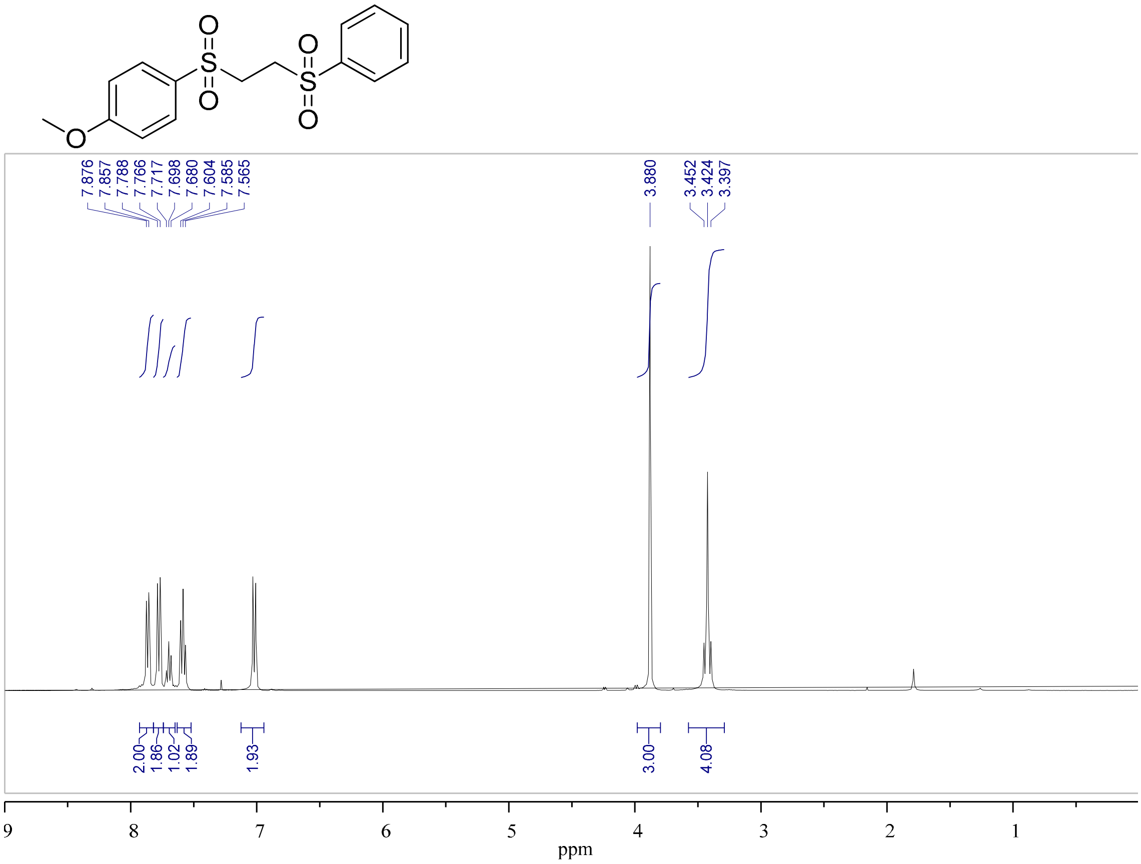


**Figure S23.** $^{1}$H-NMR spectrum of **3cg** (300 MHz, $CDCl_3$).

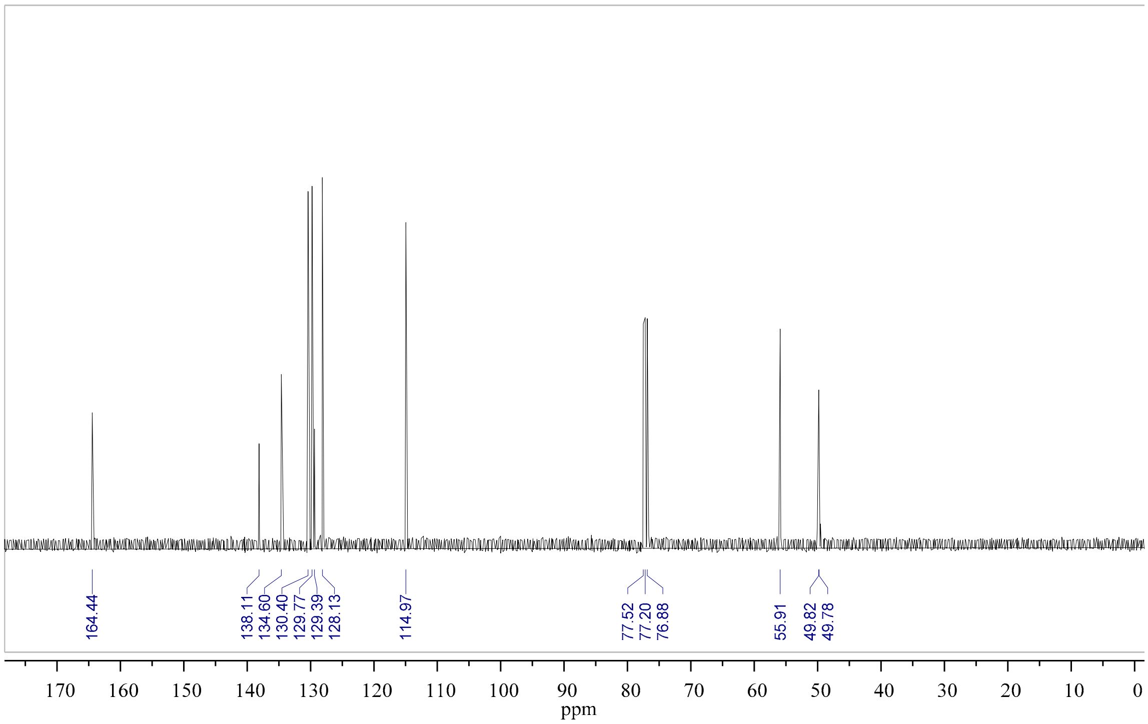


**Figure S24.** $^{13}$C-NMR spectrum of **3cg** (75 MHz, $CDCl_3$).

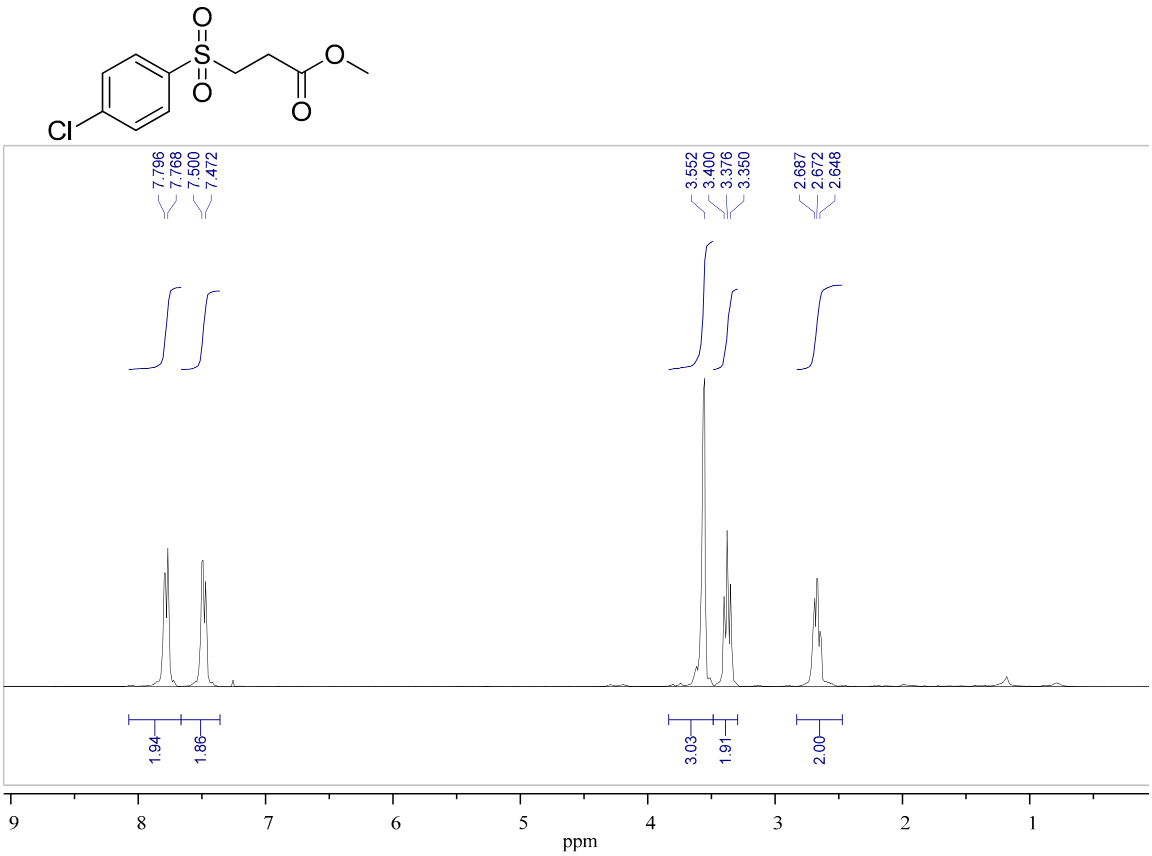


**Figure S25.** $^{1}$H-NMR spectrum of **3da** (300 MHz, $CDCl_3$).

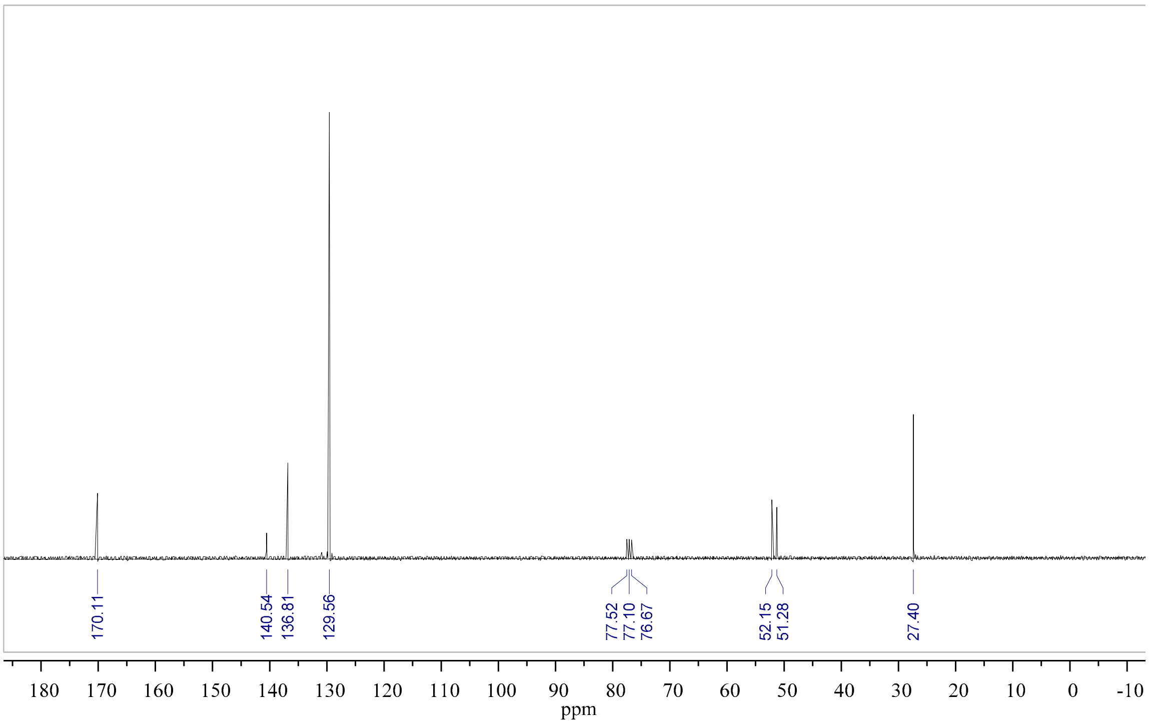


**Figure S26.** $^{13}C$-NMR spectrum of **3da** (75 MHz, $CDCl_3$).

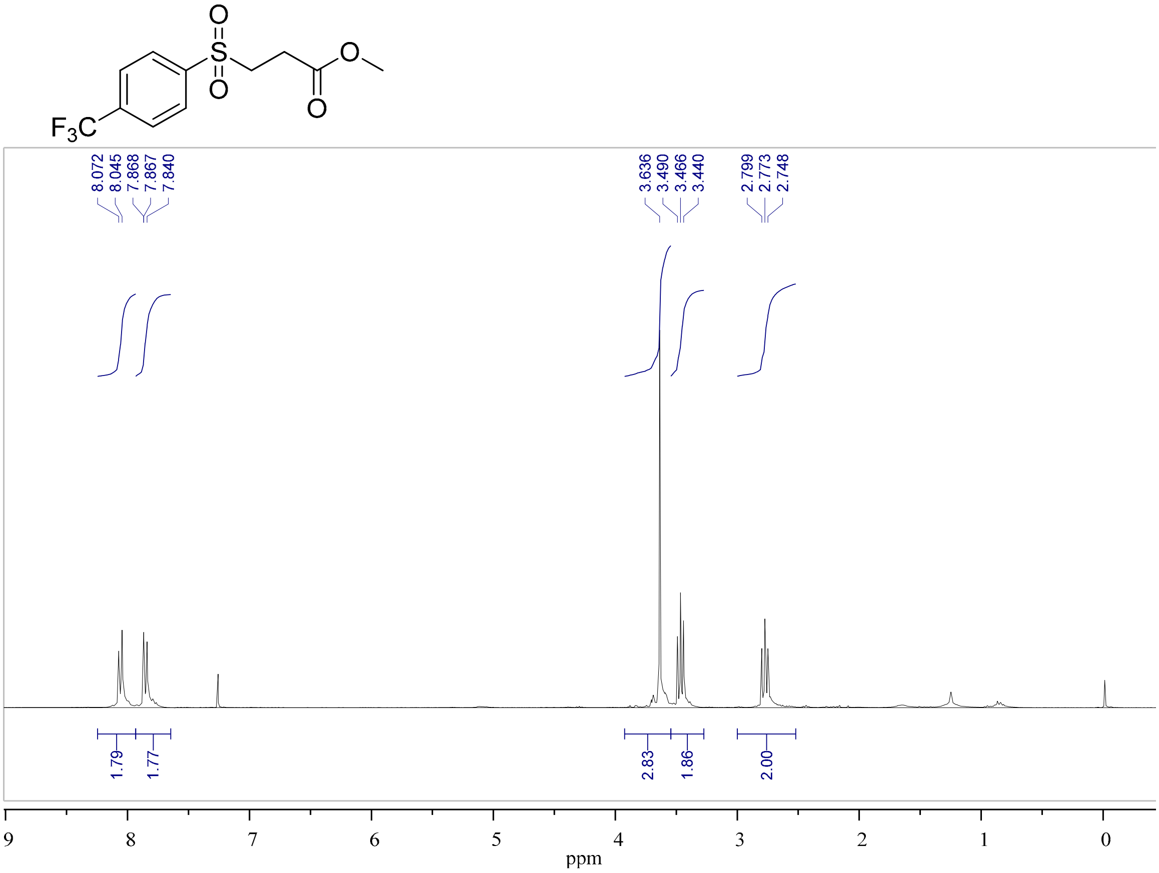


**Figure S27.** $^{1}H$-NMR spectrum of **3ea** (300 MHz, $CDCl_3$).

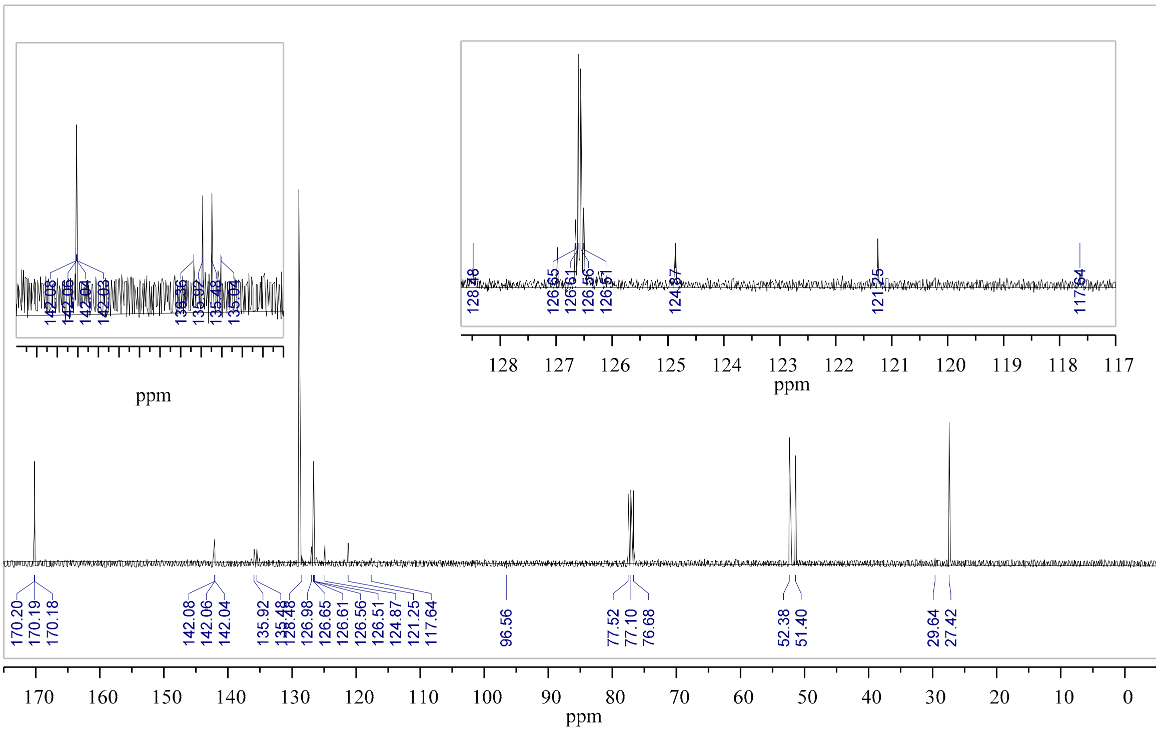


**Figure S28.** $^{13}$C-NMR spectrum of **3ea** (75 MHz, $CDCl_3$).
